# Sequelae of Severe Acute Respiratory Syndrome Coronavirus 2 (SARS-CoV-2) Infection among Kidney Transplant Recipients: A Large Single-Center Experience

**DOI:** 10.1155/2024/7140548

**Published:** 2024-05-02

**Authors:** Emily E. Zona, Mina L. Gibes, Asha S. Jain, Juan S. Danobeitia, Jacqueline Garonzik-Wang, Jeannina A. Smith, Didier A. Mandelbrot, Sandesh Parajuli

**Affiliations:** ^1^Division of Nephrology, Department of Medicine, University of Wisconsin Health, Madison, WI, USA; ^2^Baylor University Medical Center, Dallas, Texas, USA; ^3^Division of Transplantation, Department of Surgery, University of Wisconsin School of Medicine and Public Health, Madison, WI, USA; ^4^Department of Infectious Disease, University of Wisconsin Hospital and Clinics, Madison, Wisconsin, USA

## Abstract

**Background:**

Kidney transplant recipients (KTRs) are a vulnerable immunocompromised population at risk of severe COVID-19 disease and mortality after SARS-CoV-2 infection. We sought to characterize the post-infection sequelae in KTRs at our center.

**Methods:**

We studied all adult KTRs (with a functioning allograft) who had their first episode of SARS-CoV-2 infection between 04/2020 and 04/2022. Outcomes of interest included risk factors for hospitalization, all-cause mortality, COVID-19-related mortality, and allograft failure.

**Results:**

Of 979 KTRs with SARS-CoV-2 infection, 381 (39%) were hospitalized. In the multivariate analysis, risk factors for hospitalization included advanced age/year (HR: 1.03, 95% CI: 1.02–1.04), male sex (HR: 1.29, 95% CI: 1.04–1.60), non-white race (HR: 1.48, 95% CI: 1.17–1.88), and diabetes as a cause of ESKD (HR: 1.77, 95% CI: 1.41–2.21). SARS-CoV-2 Vaccination was associated with decreased risk of hospitalization (HR: 0.73, 95% CI: 0.59–0.90), all-cause mortality (HR: 0.52, 95% CI: 0.37–0.74), and COVID-19-related mortality (HR: 0.47, 95% CI: 0.31–0.71) in the univariate and multivariate analyses. Risk factors for both all-cause and COVID-19-related mortality in the multivariate analyses included advanced age, hospitalization, and respiratory symptoms for hospital admission. Furthermore, additional risk factors for all-cause mortality in the multivariate analysis included being a non-white recipient and diabetes as a cause of ESKD, with being a recipient of a living donor as protective.

**Conclusions:**

Hospitalization due to COVID-19-associated symptoms is associated with increased mortality. Vaccination is a protective factor against hospitalization and mortality.

## 1. Background

Since the beginning of the coronavirus disease 2019 (COVID-19) pandemic in December of 2019, the death rate in the United States rose by 17% from 715 deaths per 100,000 in 2019 to 835 per 100,000 in 2020 [1]. COVID-19 was the 3^rd^ leading cause of death in 2020, causing 250,831 deaths in the United States [1]. According to the World Health Organization (WHO), as of September 2023 there have been 770,778,396 confirmed cases of COVID-19, resulting in 6,958,499 deaths worldwide [2]. COVID-19 disproportionately impacts vulnerable populations, such as solid organ transplant recipients (SOTRs), who are at high risk of infection and adverse outcomes including hospitalization, allograft failure, and mortality [3]. Early studies in kidney transplant recipients with COVID-19 showed a mortality rate of approximately 30% [4]. In a national study of mortality among SOTRs during COVID‐19, there were 5083 deaths in the United States in the first 13 months of the pandemic, representing 1 in 75 SOTRs. Notably, of these deaths, kidney recipients had the highest mortality among SOTRS [5].

A multitude of risk factors have been associated with poor outcomes among KTRs with COVID-19; however, some of these risk factors are inconsistently identified or contradictory across studies. Advanced age remains an important factor in determining the risk of severe disease and mortality, and this has been widely consistent [6, 7]. The presence of other comorbidities such as diabetes mellitus, hypertension, chronic kidney disease, morbid obesity, coronary heart disease, chronic lung disease, and non-white races has been identified as major risk factors for severe disease in the general population [8, 9]. However, in one cohort study among 600 SOTRs, outcomes were not different based on reported race of the recipient, diabetes, and number/type of immunosuppressive medications were not associated with increased risk of mortality [10]. Further study is necessary to help risk stratify risk in these vulnerable patient populations.

To investigate this further, we sought to explore outcomes of the first COVID-19 infection among KTRs at our high-volume, large transplant center. Furthermore, we explored a multitude of patient-related factors and their impact on post-COVID-19 outcomes, including hospitalization and mortality.

## 2. Methods

### 2.1. Patients

We evaluated all adult (>18 years) kidney-only transplant recipients at the University of Wisconsin with longitudinal follow-up in our outpatient transplant clinic who were subsequently diagnosed with SARS-CoV-2 virus infection between April 2020 and April 2022. We excluded individuals who were followed at our center but did not have a functioning allograft. We chose April 2020 as that was when the first case of the SARS-CoV-2 virus was isolated in our kidney transplant recipient population. All recipients had at least one year of follow-up unless they had allograft failure or death. Multiorgan transplant recipients or recipients ≤18 years old at the time of diagnosis of COVID-19 were excluded. Recipients were divided into two groups based on whether they were hospitalized within the first 10 days of the diagnosis of SARS-CoV-2 infection or not. Risk factors for hospitalization, all-cause mortality, and COVID-19-related mortality at the last follow-up were outcomes of interest. We also assessed changes in allograft function at various periods post-SARS-CoV-2 infection. This study was approved by the University of Wisconsin School of Medicine and Public Health Institutional Review Board (IRB protocol number: 2014-1072). This study was in adherence to the Declaration of Helsinki. The clinical and research activities being reported were consistent with the Principles of the Declaration of Istanbul as outlined in “The Declaration of Istanbul on Organ Trafficking and Transplant Tourism.”

We follow our kidney transplant recipients at either the University Hospital or various regional outreach clinics at least once a year until graft failure or until the patient decides to transfer their care to a different center as previously described [11]. All major health events, including SARS-CoV-2 infection diagnosed at outside centers, are documented in our master database and were included in the study. Even when recipients were admitted at outside centers, in the majority of cases transplant providers at our center were consulted for management guidance.

### 2.2. Definition of Variables

We defined hospitalization, as, at the minimum, an overnight stay in the hospital. The hospitalization was considered COVID-19-related if the hospitalization was related to respiratory symptoms per initial history and physical examination. Other hospitalizations due to various indications where SARS-CoV-2 infection was diagnosed incidentally were defined as non-COVID-19-related hospitalizations. Similarly, all COVID-19-related deaths were defined if COVID-19 or SARS-CoV-2 infection was documented as a cause of mortality. All deaths were deaths with functional grafts. Death-censored graft failure (DCGF) was defined as the return to dialysis or re-transplant. All rejections were biopsy-proven acute rejection. Recipients who received at least one dose of an available SARS-CoV-2 vaccine were considered vaccinated.

### 2.3. Immunosuppression

Most KTRs at our center are maintained on a triple immunosuppressant regimen predominantly consisting of tacrolimus, mycophenolic acid, and prednisone as previously described [12]. A minority of KTRs underwent early steroid withdrawal or alternative regimens based on individual KTRs post-transplant course. At our center, we have a similar immunosuppression protocol between living or deceased donor kidney transplant recipients. At our center, we have utilized all available induction immunosuppressive agents, starting from steroid-only induction, followed by anti-lymphocyte globulin, anti-thymocyte globulin, OKT3, daclizumab or basiliximab, and recently either rabbit anti-thymocyte globulin or alemtuzumab [13].

### 2.4. Clinical Management of SARS-CoV-2 Infection

We recommend that all KTRs notify us as soon as they test positive or are suspected of having COVID-19. Although not formally protocolized, antimetabolites were decreased by 25–50% for the first 2-3 weeks after diagnosis of SARS-CoV-2 infection for mild to moderate symptoms. Also, we recommend that all recipients seek early available treatment for COVID-19 if they tested positive. We also recommend that patients seek urgent local evaluation if their oxygen saturation drops below 90%, if they experience worsening shortness of breath, inability to hydrate due to vomiting or diarrhea, or alteration in mentation. During the study period, all patients were tested for SARS-CoV-2 before being admitted to the hospital.

### 2.5. Statistical Analysis

Continuous data were compared using Student's *t*-test or the Wilcoxon rank-sum test, as appropriate, while categorical data were analyzed using Fisher's exact test or chi-square test. *P* values ≤0.05 were considered statistically significant. Risk factors associated with hospitalization, all-cause mortality, and COVID-19-related mortality were studied using univariate and multivariate stepwise Cox regression analyses. Some of the baseline characteristics were used to assess the risk. Variables associated with outcomes at a *P* value ≤0.10 in univariate analysis were kept in the multivariate analysis. All-cause mortality and COVID-19-related mortality were analyzed using Kaplan–Meier analyses.

## 3. Results

We identified 979 kidney transplant recipients with a SARS-CoV-2 infection diagnosis, of which 381 (39%) required hospitalization, whereas 598 (61%) were treated in the outpatient setting. These recipients were transplanted between 10/1978 and 01/2021. In the initial year spanning from April 2020 to April 2021, 346 (35%) presented with a positive SARS-CoV-2 infection diagnosis. From this subset, 144 (42%) underwent hospitalization, while 202 (58%) received non-hospital-based care. Similarly, from May 2021 to April 2022, 633 (65%) presented with a positive SARS-CoV-2 infection diagnosis. From this subset, 237 (37%) underwent hospitalization, while 396 (63%) received non-hospital-based care. The rate of hospitalization between the two eras of 42% vs 37% was not statistically significantly different (*P*=0.20).

Among the entire cohort, there were some differences in the baseline characteristics between those hospitalized and those not hospitalized, as summarized in Table 1. At the time of the SARS-CoV-2 infection diagnosis, recipients requiring hospitalization were older, more likely to be male and, non-white, more likely to have diabetes as the cause of end-stage kidney disease (ESKD), and were more likely to be recipients of deceased donor allografts. Similarly, recipients requiring hospitalization were likely to be maintained on a standard triple immunosuppressive regimen including prednisone-based immunosuppression and had inferior graft function at baseline as assessed by estimated glomerular filtration rate (eGFR).

Among the 381 recipients who required hospitalization, 96 (25%) were admitted to our university hospital, while the remainder received care at various other medical facilities. The most common indication for hospitalization was COVID-19-related respiratory symptoms, found in 285 (75% of hospitalized) recipients. Other common indications for admission were weakness/malaise/change in mental status in 16 (4%) recipients, gastrointestinal symptoms in 14 (4%) recipients, and acute kidney injury in 11 (3%). The remaining had various other indications including incidental diagnosis without symptoms. Of 381 recipients requiring hospitalization, 88 (23%) were admitted to the intensive care unit (ICU), as outlined in Table 2. Graft function, only among those with only functional graft (censored at death or graft failure), was inferior among the hospitalized group, from the baseline to the last follow-up (Supplementary Figure 1). At last follow-up, 205 recipients (21%) experienced uncensored graft failures, of which 156 (16%) resulted from death. Notably, 119 (12%) of these deaths were primarily attributed to COVID-19, with the majority occurring in the hospitalized group.

Assessing the risk for hospitalization (Table 3) in univariate analysis, older age at the time of SARS-CoV-2 infection diagnosis, male recipients, non-white race, diabetes as a cause of ESKD, triple standard immunosuppressive medications, and prednisone-based immunosuppression were associated with increased risk for hospitalization. Meanwhile, living-donor recipients and higher baseline eGFR were associated with lower risk for hospitalization. In the multivariate analysis, older age (HR: 1.03; 95% CI: 1.02–1.04; *P* < 0.001); male gender (HR: 1.29; 95% CI: 1.04–1.60; *P*=0.02); non-white race (HR: 1.48; 95% CI: 1.17–1.88; *P*=0.001); and diabetes as a cause of ESKD (HR: 1.77; 95% CI: 1.41–2.21; *P* < 0.001) were still associated with increased risk for hospitalization, while vaccination against SARS-CoV-2 infection (HR: 0.73; 95% CI: 0.59–0.90; *P*=0.003) and higher baseline eGFR (HR: 0.98; 95% CI: 0.97–0.99; *P* < 0.001) were protective.

**Figure 1:**
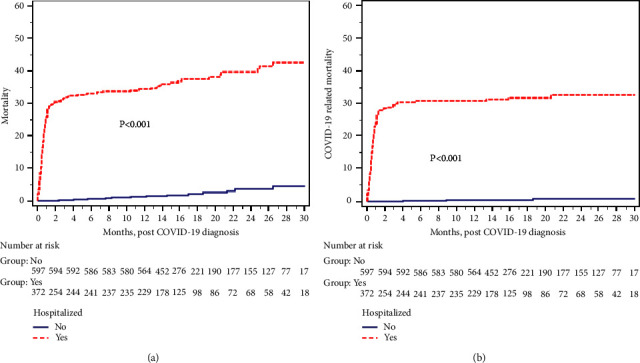
Increase in the risk of all-cause mortality (a) or COVID-19-related mortality (b) among kidney transplant recipients needing hospitalization mainly within a few months of the infection.

Similarly, looking at the risk of all-cause mortality (Table 4) in univariate analysis, older age, male recipient, non-white recipient, diabetes as the case of ESKD, prednisone-based immunosuppression, the longer interval from transplant to SARS-CoV-2 infection diagnosis, and respiratory symptoms for hospital admission were associated with increased risk. Hospitalization therefore was also significantly associated with an increased risk of all-cause mortality, mainly within the early post-SARS-CoV-2 infection interval, as shown in Figure 1(a). Living donor recipients, vaccination against SARS-CoV-2 infection, and higher baseline eGFR were protective factors. In multivariate analysis, older age (HR: 1.05; 95% CI: 1.03–1.07; *p* < 0.001); non-white recipient (HR: 1.46; 95% CI: 1.01–2.12; *P*=0.04); diabetes as a cause of ESKD (HR: 1.77; 95% CI: 1.41–2.21; *p* < 0.001); the longer interval from transplant to the COVID-19 diagnosis (HR: 1.01; 95% CI: 1.0–1.01; *P*=0.008); hospitalization (HR: 6.76; 95% CI: 3.43–13.28; *P* < 0.001); and respiratory symptoms for hospital admission (HR: 2.29; 95% CI: 1.42–3.68; *P*<0.001) were associated with increased risk of death. Living donor recipient (HR: 0.69; 95% CI: 0.48–1.0; *P*=0.049); vaccination against COVID-19 (HR: 0.52; 95% CI: 0.37–0.74; *P* < 0.003); and higher baseline eGFR (HR: 0.98; 95% CI: 0.97–1.0; *P*=0.01) were protective.

Similar outcomes were found when looking at the COVID-19-related mortality risk in Table 5 and Figure 1(b). In multivariate analysis, the older the recipient age, the longer the interval from transplant to the COVID-19 diagnosis, hospitalization, and respiratory symptoms for hospitalization were associated with increased risk, while vaccination against COVID-19 was protective. Male recipients, non-white recipients, diabetes as the cause of ESKD, living donor recipients, and baseline eGFR did not impact the risk for COVID-19-related mortality.

As the rate of hospitalization was significantly lower among living donor kidney recipients, these recipients also had better eGFR at 46.1 ± 21.3 mL/min/1.73 m^2^ compared to 41.7 ± 20.3 mL/min/1.73 m^2^ among deceased donors (*P*=0.03) at time of the SARS-CoV-2 infection diagnosis or even before the SARS-CoV-2 infection at 54.8 ± 20.3 mL/min/1.73 m^2^ among living donor recipients compared to 51.8 ± 21.3 mL/min/1.73 m^2^ among deceased donors (*P*=0.03).

## 4. Discussion

In this large cohort of 979 KTRs from a single center within the first two years of the COVID-19 pandemic, we identified some interesting and important features associated with SARS-CoV-2 infection and the downstream sequelae. Most of the risk factors for hospitalization, overall mortality, and COVID-19-related mortality in our cohort were non-modifiable, including older age of the recipient, male gender, non-white race, diabetes, and suboptimal graft function. However, the most important and striking findings were the protective effects of COVID-19 vaccination for hospitalization, overall mortality, and COVID-19-related mortality in this cohort. Our findings are consistent with previous studies of the protective effect of vaccination in SOTRs [14–17]. These findings reiterate the importance of vaccination against COVID-19 in kidney transplant recipients. Also, likely due to the better allograft function, living donors had protective effects against hospitalization or all-cause mortality.

Multiple studies have found that immunocompromised patients, including SOTRs, are at higher risk of hospitalization or death [18, 19]. It has been widely reported that older age and prolonged intervals from transplant remain important factors in determining the risk of severe disease and mortality [6, 7]. There is also substantial evidence demonstrating that gender is an important driver of the risk of mortality and response to SARS-CoV-2 infection, and multiple studies have shown that men tend to have more severe disease and higher mortality from COVID-19 [20–22]. Differences in the innate immune response, sex hormone biology, and angiotensin-converting enzyme 2 expression have been suggested as the underlying pathophysiological mechanisms behind these observations [23]. In addition to all the traditional risk factors, SOTRs are also at increased risk for more severe disease and mortality due to the necessity of various immunosuppressive agents to prevent the allograft from rejection [24, 25].

SARS-CoV-2 vaccination remains the most effective modifiable way to prevent serious outcomes and death from SARS-CoV-2 infection. By December 2020, within the first year of the pandemic, mass vaccination programs were initiated [26]. Additionally, by November 2021, 7 vaccines had received WHO Emergency Use Listing, including the mRNA vaccines BNT162b2 (Pfizer/BioNTech) and mRNA-1273 (Moderna), viral vector vaccines ChAdOx1 nCoV-19 (Oxford/AstraZeneca), Ad26.COV2.S (Johnson & Johnson), and others [26]. Although SOTRs were prioritized for vaccination, vaccine efficacy among these populations was not tested before rollout [27]. Later, it was recognized that immunological responses to SARS-CoV-2 vaccines were lower in SOTRs compared to the general population. After the first dose, antibody responses were detected in just 6–17% of SOTRs, and cellular responses were observed in around 25%, and that response rose to 18–64% for antibody response and 30–79% for the cellular response after the second dose [26]. T-cell responses can occur even without detectable antibody responses, and thus patients without antibodies could still mount a sufficient immune response to prevent severe infection. As such, the presence of antibodies should not be interpreted as indicating “immune protection” and routine antibody monitoring after vaccination is not universally recommended [26]. Studies have similarly shown improvements in antibody and T-cell responses after the third dose, including improved serum neutralizing capacity and a rise in antibody titers in previously seropositive patients, along with 30–50% seroconversion from negative to positive among recipients who were seronegative even after second doses [26]. The limited vaccine effectiveness even for the boosters has been a concern. However, similar to the initial vaccine series, boosters have been associated with preventing critical COVID-19-associated outcomes including ICU admission and death [28].

Despite the reduction in disease severity due to the availability of the COVID-19 vaccination series, infections continue to be linked with adverse outcomes, including the need for mechanical ventilation, the development of acute respiratory distress syndrome, and an increased risk of acute kidney injury [29–32]. COVID-19 infection has been reported to cause vasculitis and vasculopathy and could mimic allograft rejection [33]. Also, in our own experience, we noticed unusually high rates of acute rejection during the pandemic [34]. Although allograft biopsy is the gold standard for the diagnosis of rejection, we reported the utility of donor-derived cell-free DNA for assessment of rejection in these patients during pandemics [35].

This study has the expected limitations of a single-center observational study, reflecting our specific population and clinical approach. Our findings are reflective of the practices at our center, and this should be factored into the interpretation. However, this substantial dataset with more granular data provides useful information for estimating risks and outcomes. Also, to the best of our knowledge, this study is the largest of its kind, with 979 kidney transplant recipients from a single center with prospective follow-up. Also, as management and vaccination protocols during the study period were evolving, it was not possible to provide the details of the management of COVID-19 infection or vaccination protocols. Furthermore, we did not analyze the data based on the different waves and variants of COVID-19. Also, we did not analyze the outcomes, based on the induction immunosuppressive agents, as in this cohort, we had recipients transplanted at various timeframes with mean intervals from transplant to COVID-19 diagnosis of more than 89 months, which could lower the correlation between induction and various outcomes of interest.

In summary, COVID-19 has had a long-lasting impact on health outcomes of patients living with kidney transplants. Continued efforts to define rigorous medical and psychosocial patient-centered, risk stratification strategies are necessary to avoid adverse outcomes [36]. In May 2023, WHO declared “with great hope” an end to COVID-19 as a public health emergency, stressing that it does not mean the disease is no longer a global threat [37]. However, in clinical practice we are still seeing patients suffering from COVID-19, particularly SOTRs. Based on our data, we believe vaccine uptake is important in our patients who are the most vulnerable to the negative impact of the disease.

## Figures and Tables

**Table 1:** Baseline characteristics.

Characteristics			All (*n* = 979)	Hospitalized (*n* = 381)	Non-hospitalized (*n* = 598)	*P*
Age at transplant (yrs)			49.3 ± 13.4	52.4 ± 13.4	47.3 ± 13.0	**<0.001**

Age at COVID-19 diagnosis (yrs)			56.7 ± 12.9	59.8 ± 12.9	53.7 ± 12.6	**<0.001**

Male, %			567 (58)	238 (63)	329 (55)	**0.02**

Non-white, %			205 (21)	108 (28)	97 (16)	**<0.001**

Cause of ESKD, %		Diabetes mellitus	210 (22)	124 (33)	86 (14)	**<0.001**
		Hypertension	114 (12)	51 (13)	63 (11)	
		Glomerular disease	307 (31)	102 (27)	205 (34)	
		Polycystic kidney disease	137 (14)	35 (9)	102 (17)	
		Other	211 (22)	69 (18)	142 (24)	

Living donor, %			417 (43)	133 (35)	284 (48)	**<0.001**

Previous transplant, %			201 (21)	74 (19)	127 (21)	0.49

Maintenance immunosuppressive, %	Tacrolimus + mycophenolic acid + prednisone		615 (63)	256 (67)	359 (60)	**0.02**
	Prednisone based immunosuppression		858 (88)	347 (91)	511 (86)	**0.009**

Vaccinated (%)			436 (45)	156 (41)	280 (47)	0.07

Rejection within six months prior to COVID-19 (%)			10 (1)	4 (1)	6 (1)	0.94

Baseline serum creatinine before COVID-19 (mg/dl)			1.52 ± 0.76	1.71 ± 0.97	1.41 ± 0.56	**<0.001**

Baseline eGFR before COVID-19 (mL/min/1.73 m^2^)			53.1 ± 20.9	47.1 ± 19.8	57.0 ± 20.8	**<0.001**

Interval from transplant to the first COVID-19 (mo)			89.5 ± 85.5	89.5 ± 85.8	89.5 ± 85.4	0.98

Bold values signify statistically significant with *p* value less than 0.05.

**Table 2:** Outcomes.

Characteristics	All	Hospitalized	Non-hospitalized	*P*
	979	381	598	
Respiratory symptoms for admission (%)	285 (29)	285 (75)	N/A	
Intensive care unit admission (%)	88 (9)	88 (23)	NA	
Serum creatinine at the time of SARS-CoV-2 infection (mg/dl)	2.01 ± 1.80	2.25 ± 1.56	1.59 ± 0.61	**<0.001**
Serum eGFR at the time of SARS-CoV-2 infection (mL/min/1.73 m^2^)	43.4 ± 20.8	39.6 ± 20.8	50.2 ± 18.9	**<0.001**
Serum creatinine 1 month post SARS-CoV-2 infection (mg/dl)	1.53 ± 0.83	1.70 ± 1.05	1.43 ± 0.64	**<0.001**
Serum eGFR 1 month post SARS-CoV-2 infection (mL/min/1.73 m^2^)	54.6 ± 22.8	49.8 ± 23.0	57.3 ± 22.3	**<0.001**
Serum creatinine 6 months post SARS-CoV-2 infection (mg/dl)	1.51 ± 0.74	1.69 ± 0.92	1.43 ± 0.63	**<0.001**
Serum eGFR 6 months post SARS-CoV-2 infection (mL/min/1.73 m^2^)	55.5 ± 22.2	49.9 ± 22.0	58.0 ± 21.8	**<0.001**
Serum creatinine 1 year post SARS-CoV-2 infection (mg/dl)	1.52 ± 0.88	1.74 ± 1.14	1.42 ± 0.71	**<0.001**
Serum eGFR 1 year post SARS-CoV-2 infection (mL/min/1.73 m^2^)	56.6 ± 22.8	49.9 ± 22.1	59.4 ± 22.5	**<0.001**
Serum creatinine at last follow-up (mg/dl)	1.47 ± 0.71	1.63 ± 0.89	1.41 ± 0.62	**<0.001**
Serum eGFR at last follow-up (mL/min/1.73 m^2^)	57.8 ± 22.6	52.9 ± 22.5	59.6 ± 22.5	**<0.001**
Uncensored graft failure at last follow-up (%)	205 (21)	170 (45)	35 (6)	**<0.001**
Death at last follow-up (%)	156 (16)	141 (37)	15 (3)	**<0.001**
Death related to COVID-19 (%)	119 (12)	116 (30)	3 (1)	**<0.001**

Bold values signify statistically significant with *p* value less than 0.05.

**Table 3:** Risk factors for hospitalization.

Covariate	Univariate analyses			Multivariate analyses		
	HR	95% CI	*P*	HR	95% CI	*P*
Age at SARS-CoV-2 infection diagnosis (per year)	1.03	1.02–1.04	**<0.001**	1.03	1.02–1.04	**<0.001**
Male recipient	1.29	1.05–1.59	**0.01**	1.29	1.04–1.60	**0.02**
Non-white recipient	1.59	1.27–1.99	**<0.001**	1.48	1.17–1.88	**0.001**
Diabetes as a cause of ESKD vs other	2.05	1.66–2.55	**<0.001**	1.77	1.41–2.21	**<0.001**
Living donor recipient	0.69	0.56–0.85	**0.006**	0.91	0.72–1.13	0.40
Previous transplant	0.89	0.68–1.15	0.36			
Tacrolimus + MPA + prednisone maintenance vs other	1.28	1.03–1.59	**0.02**	1.18	0.92–1.51	0.18
Prednisone-based immunosuppression	1.80	1.26–2.58	**0.001**	1.29	0.86–1.95	0.22
Treatment of rejection before SARS-CoV-2 infection	0.97	0.36–2.62	0.97			
Vaccinated	0.81	0.66–0.99	**0.04**	0.73	0.59–0.90	**0.003**
Baseline eGFR pre-SARS-CoV-2 infection (per mL/min/1.73 m^2^)	0.98	0.97–0.99	**<0.001**	0.98	0.97–0.99	**<0.001**
Interval from transplant to COVID-19 (per month)	1.01	0.99–1.02	0.19			

Bold values signify statistically significant with *p* value less than 0.05.

**Table 4:** Risk for all-cause mortality.

Covariate	Univariate analyses			Multivariate analyses		
	HR	95% CI	*P*	HR	95% CI	*P*
Age at COVID-19 diagnosis (per year)	1.07	1.06–1.09	**<0.001**	1.05	1.03–1.07	**<0.001**
Male recipient	1.57	1.12–2.19	**0.009**	1.26	0.89–1.78	0.19
Non-white recipient	1.76	1.24–2.48	**0.001**	1.46	1.01–2.12	**0.04**
Diabetes as a cause of ESKD vs other	2.54	1.84–3.50	**<0.001**	1.42	1.0–2.01	**0.04**
Living donor recipient	0.51	0.36–0.73	**0.002**	0.69	0.48–1.0	**0.049**
Previous transplant	0.65	0.42–1.01	0.06	1.06	0.66–1.69	0.80
Tacrolimus + MPA + prednisone maintenance vs other	1.04	0.75–1.45	0.79			
Prednisone-based immunosuppression	2.80	1.38–5.72	**0.004**	1.45	0.69–3.06	0.96
Treatment of rejection before SARS-CoV-2 infection	0.45	0.1–1.79	0.26			
Vaccinated	0.53	0.37–0.74	**<0.001**	0.52	0.37–0.74	**0.003**
Baseline eGFR (per mL/min/1.73 m^2^)	0.98	0.97–0.99	**<0.001**	0.98	0.97–1.0	**0.01**
Interval from transplant to COVID-19 (per month)	1.01	1.0–1.03	**0.03**	1.01	1.0–1.01	**0.008**
Hospitalization	19.18	11.7–32.7	**<0.001**	6.76	3.43–13.28	**<0.001**
Respiratory symptoms for hospital admission	11.31	7.5–16.5	**<0.001**	2.29	1.42–3.68	**<0.001**

Bold values signify statistically significant with *p* value less than 0.05.

**Table 5:** Risk for COVID-19-related mortality.

Covariate	Univariate analyses			Multivariate analyses		
	HR	95% CI	*P*	HR	95% CI	*P*
Age at SARS-CoV-2 infection diagnosis (per year)	1.06	1.05–1.08	**<0.001**	1.04	1.02–1.05	**<0.001**
Male recipient	1.82	1.22–2.69	**0.003**	1.39	0.93–2.08	0.11
Non-white recipient	1.82	1.23–2.69	**0.002**	1.45	0.94–2.21	0.09
Diabetes as a cause of ESKD vs other	2.41	1.67–3.50	**<0.001**	1.36	0.91–2.01	0.13
Living donor recipient	0.58	0.39–0.85	**0.005**	0.77	0.51–1.16	0.21
Previous transplant	0.54	0.32–0.93	**0.03**	0.94	0.53–1.66	0.83
Tacrolimus + MPA + prednisone maintenance vs other	1.05	0.73–1.53	0.78			
Prednisone-based immunosuppression	2.37	1.11–5.09	**0.03**	1.28	0.57–2.86	0.55
Treatment of rejection before SARS-CoV-2 infection	0.89	0.13–6.24	0.89			
Vaccinated	0.44	0.29–0.5	**<0.001**	0.47	0.31–0.71	**<0.001**
Baseline eGFR (per mL/min/1.73 m^2^)	0.98	0.97–0.99	**<0.001**	0.99	0.98–1.01	0.18
Interval from transplant to SARS-CoV-2 infection (per month)	1.02	1.0–1.04	**0.007**	1.01	1.0–1.05	**0.003**
Hospitalization	76.2	24.2–239.7	**<0.001**	24.3	6.9–85.7	**<0.001**
Respiratory symptoms for hospital admission	20.0	11.80–33.88	**<0.001**	2.73	1.52–4.89	**<0.001**

Bold values signify statistically significant with *p* value less than 0.05.

## Data Availability

The data used to support the findings of this study are available from the corresponding author upon reasonable request.

## Abbreviations

COVID-19: Coronavirus disease 2019
eGFR: Estimated glomerular filtration rate
ESKD: End-stage kidney disease
KTRs: Kidney transplant recipients
SARS-CoV-2: Severe acute respiratory syndrome coronavirus 2
SOTRs: Solid organ transplant recipients
WHO: World Health Organization.

## Supplementary Materials

Supplementary Materials Supplemental Figure 1: changes in eGFR among those needing hospitalization and not needing hospitalization at various time frames after COVID-19 infection, censored at death or graft failure.
