# Supplementary figures and images for: Sequelae of Severe Acute Respiratory Syndrome Coronavirus 2 (SARS-CoV-2) Infection among Kidney Transplant Recipients: A Large Single-Center Experience
Source: Crit Care Res Pract. 2024 May 2;2024:7140548. doi: 10.1155/2024/7140548 (PMC11081755; doi:10.1155/2024/7140548)

Supplemental Figure 1: Changes in eGFR

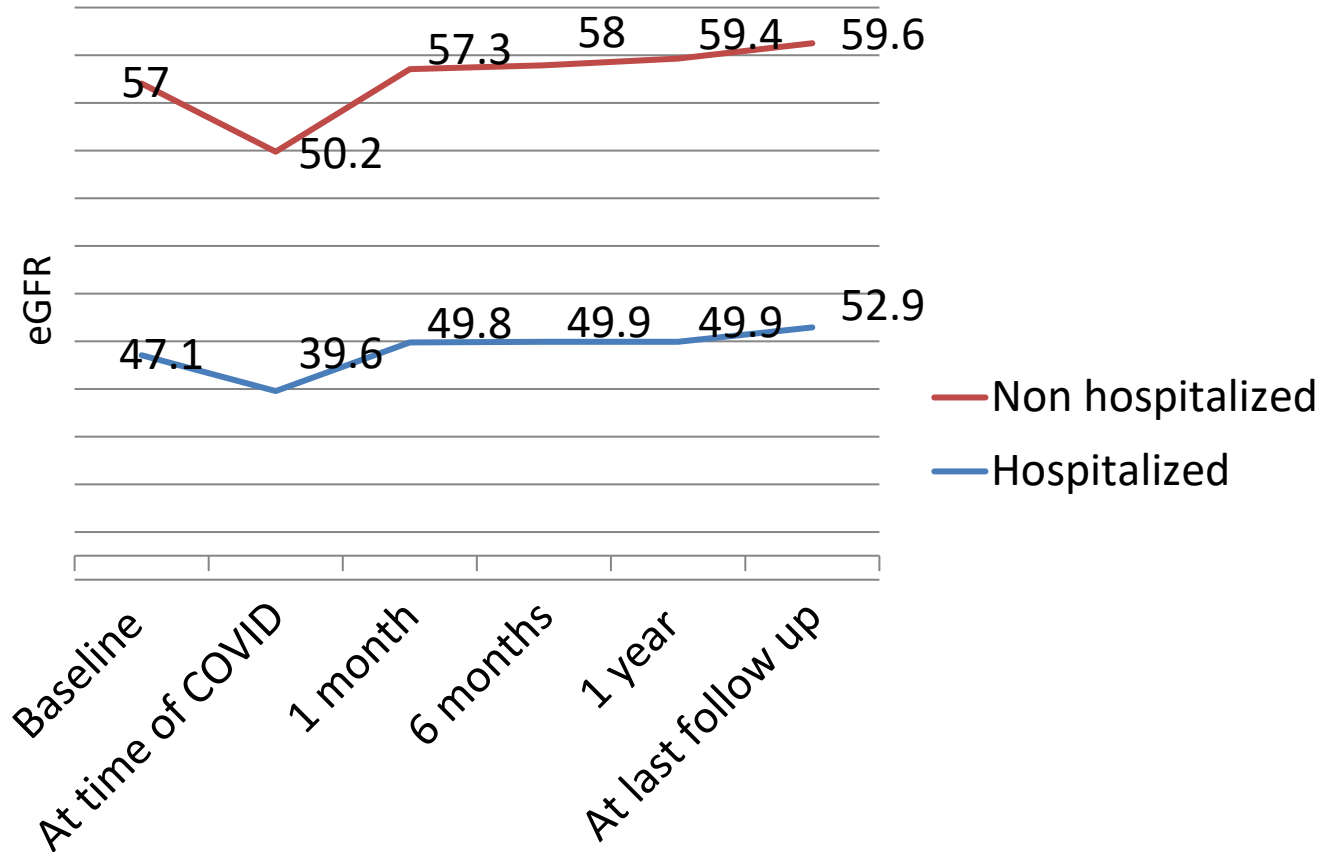

Supplement: Supplementary Materials — Supplemental Figure 1: changes in eGFR among those needing hospitalization and not needing hospitalization at various time frames after COVID-19 infection, censored at death or graft failure. [file 7140548.f1.pdf]
